# A 23-year study of mortality and development of co-morbidities in patients with obesity undergoing bariatric surgery (laparoscopic gastric banding) in comparison with medical treatment of obesity

**DOI:** 10.1186/s12933-018-0801-1

**Published:** 2018-12-29

**Authors:** Antonio E. Pontiroli, Ahmed S. Zakaria, Marco Fanchini, Chiara Osio, Elena Tagliabue, Giancarlo Micheletto, Alessandro Saibene, Franco Folli

**Affiliations:** 1 Università degli Studi di Milano, Milan, Italy; 2 ASST Santi Paolo e Carlo, Milan, Italy; 3 Istituto Multimedica, Milan, Italy; 4 INCO-Istituto Clinico Sant’Ambrogio, Milan, Italy; 5 Ospedale San Raffaele, Milan, Italy

**Keywords:** Bariatric surgery, Survival, Adjustable gastric banding, Diabetes mellitus, Cancer, Cardiovascular disease, Exemptions, Hospital admissions, Obesity, Mortality, Prevention of diabetes, Prevention of cardiovascular disease, ICD10, Kaplan–Meier, Cox proportional hazards model

## Abstract

**Background and aim:**

Several studies have shown that bariatric surgery reduces long term mortality compared to medical weight loss therapy. In a previous study we have demonstrated that gastric banding (LAGB) is associated with reduced mortality in patients with and without diabetes, and with reduced incidence of obesity co-morbidities (cardiovascular disease, diabetes, and cancer) at a 17 year follow-up. The aim of this study was to verify at a longer time interval (23 years) mortality and incidence of co-morbidities in patients undergoing LAGB or medical weight loss therapy.

**Patients and methods:**

As reported in the previous shorter-time study, medical records of obese patients [body mass index (BMI) > 35 kg/m^2^ undergoing LAGB (n = 385; 52 with diabetes) or medical treatment (controls, n = 681; 127 with diabetes), during the period 1995–2001 (visit 1)] were collected. Patients were matched for age, sex, BMI, and blood pressure. Identification codes of patients were entered in the Italian National Health System Lumbardy database, that contains life status, causes of death, as well as exemptions, prescriptions, and hospital admissions (proxies of diseases) from visit 1 to June 2018. Survival was compared across LAGB patients and matched controls using Kaplan–Meier plots adjusted Cox regression analyses.

**Results:**

Final observation period was 19.5 ± 1.87 years (13.4–23.5). Compared to controls, LAGB was associated with reduced mortality [HR = 0.52, 95% CI 0.33–0.80, p = 0.003], significant in patients with diabetes [HR = 0.46, 95% CI 0.22–0.94, p = 0.034], borderline significant in patients without diabetes [HR = 0.61, 95% CI = 0.35–1.05, p = 0.076]. LAGB was associated with lower incidence of diabetes (15 vs 75 cases, p = 0.001), of CV diseases (61 vs 226 cases, p = 0.009), of cancer (10 vs 35, p = 0.01), and of renal diseases (0 vs 35, p = 0.001), and of hospital admissions (92 vs 377, p = 0.001).

**Conclusion:**

The preventive effect of LAGB on mortality is maintained up to 23 years, even with a decreased efficacy compared with the shorter-time study, while the preventive effect of LAGB on co-morbidities and on hospital admissions increases with time.

**Electronic supplementary material:**

The online version of this article (10.1186/s12933-018-0801-1) contains supplementary material, which is available to authorized users.

## Introduction

Patients with obesity undergoing bariatric surgery have a longer life expectancy than patients receiving medical treatment of obesity. Several papers [1–8], analyzed in two meta-analyses [9, 10], have shown lower long-term mortality with bariatric surgery in comparison with nonsurgical controls; further, reduced mortality is observed in patients with and without diabetes [1, 4, 11, 12]. In addition, bariatric surgery improves quality of life in morbid obesity [13], is associated with lower development of medical complications of obesity, reduced frequency of co-morbidities, improved cardiovascular (CV) risk profile [14–20], and is cost-effective in the management of obesity [21, 22]. The majority of studies has been performed through well established restrictive or mixed techniques [gastric banding (LAGB), vertical banded gastroplasty (VGB), roux-en-y gastric bypass (RYGB)], but recent studies have shown that laparoscopic sleeve gastrectomy (LSG) [23], as well as malabsorptive surgery [biliointestinal bypass (BIBP) and biliopancreatic diversion (BPD)] is associated with reduced mortality and lower development of obesity related co-morbidities, compared to medical weight loss treatment of obesity [24].

No intermediate evaluation of clinical and metabolic effects of bariatric surgery, in comparison with medical treatment of obesity, has appeared in previous studies evaluating long-term mortality, so that reduced mortality seems an all-or-none effect, with no mechanistic explanation for the reduced mortality.

In a previous retrospective study we have shown that, up to 17 years, LAGB is associated with reduced mortality in patients with and without diabetes, and with reduced incidence of diabetes and cardiovascular diseases [11]. This was the longest follow-up study, with no patient lost to follow-up; we also hypothesized that a longer follow-up was required to establish if the effects of LAGB were maintained or even made more significant through a prolonged observation, or whether the effects of LAGB vanished, also because of the process of aging.

The aim of this retrospective study was to extend the follow-up period observation of the previous study up to 23 years. In addition, we had the opportunity to compare the intermediate clinical and metabolic effects of bariatric surgery and of medical treatment of obesity, thus evaluating a possible mechanistic explanation for the reduced mortality.

## Methods

### Patients and study protocol

The participating institutions offer surgical and medical treatment of obesity. The institutions belong to the LAGB10 study group [11], a spontaneous network of physicians and surgeons working with bariatric surgery in the Lumbardy Region (Italy); LAGB has been performed here since 1995, according to NIH guidelines [25]. The specific study protocol was approved by four Ethics Committees in 2012, after the initial protocol had been approved in 1995, in 2002, and in 2006. Being a retrospective study, informed consent was obtained from all individual participants included in the study who could be reached by interview, phone or letter. The details of the protocol have been previously published [11]. Briefly, we considered all patients with obesity (BMI > 40 kg/m^2^ alone or BMI > 35 kg/m^2^ in the presence of co-morbidities) aged 18–65 years, seeking medical advice and referred to the outpatients obesity clinics during the period 1995–2001, (first visit) undergoing thereafter LAGB, or medical weight loss treatment. After evaluation of indications and contra-indications, patients were offered LAGB; several patients declined the offer, mainly because of reluctancy, lack of knowledge of the possible benefits, fear of surgery and of surgical complications, inability or unwillingness to comply with the anticipated change of lifestyle habits or with the program of scheduled visits. Patients who declined surgery for any reason, but agreed to be followed-up during medical treatment, were considered controls. All surgery and nonsurgical patients were treated with diet, and received standard care (education on eating behaviors, advice on diet and exercise, plus drug treatment for diabetes and hypertension when present). At least initially, all patients were evaluated under basal conditions and at 3-month intervals with measurement of body weight and assessment of food intake through review of diet diaries; their suggested diet was between 1000 and 1200 kcal/day for women and men (22% protein, 29% lipids, and 49% carbohydrates), respectively, with the aid of a dietitian. From the medical records, birthdate and age, baseline anthropometric data (height, weight, BMI) systolic and diastolic blood pressure, heart rate, metabolic data (fasting blood glucose, glycated hemoglobin [HbA1c (%)], total cholesterol, HDL-, and LDL-cholesterol, triglycerides, aspartate transferase [AST], alanine transferase [ALT], creatinine and eGFR [modified diet in renal disease calculation equation] [26]), current medical treatments, clinical evidence of coronary heart disease (CHD), retinopathy, were derived and tabulated. From the medical records it was also possible to evaluate later visits and lab examination, when present. Diagnosis of diabetes (type 2 diabetes) was established as already reported [27, 28], and diagnosis of coronary heart disease (CHD) was based on medical records.

### Procedures

Patients were identified through personal identification codes; codes were entered the Regional Lumbardy Administrative Database, and it was possible to ascertain whether patients were alive, were dead, or had moved to other regions. The National Health System (NHS) covers more than 95% of all hospital admissions, medical and surgical procedures and medical expenses of citizens [29] (Italian Survey 2012). The Regional Lumbardy Administrative Database contains since 1988 all pertinent data of all citizens, and this makes life status a clear finding, independently of participation in studies and of loss to follow-up. In particular, the Lumbardy database collects several information, including (1) an archive of residents who receive NHS assistance, reporting demographic and administrative data; (2) a database on diagnosis at discharge from public or private hospitals of the region; (3) a database on outpatient drug prescriptions reimbursable by the NHS; and (4) a database on outpatient visits, including visits in specialist ambulatory care and diagnostic laboratories accredited by the NHS. For each patient, these databases are linked through a single identification code.

In the Italian National Health System development of chronic diseases (diabetes mellitus, liver and cardiovascular diseases, selected thyroid, renal, and lung diseases) yields the right to exemption from medical charges (exemptions), that means life-long free prescriptions and examinations for the above diseases. Therefore, together with hospital admissions, exemptions were considered a proxy of development of chronic diseases. For each patient, exemptions and hospital admissions after first visit were identified and dated. Through registries of surgeons and the Regional Lumbardy Administrative Database it was also possible to retrieve patients who had removal of LAGB and/or new bariatric surgery procedures. Through the health districts (ASL) patients belonged to, it was possible to track causes of death, and nature of hospital admissions and of exemptions. Data from health districts were cross-checked with data from the Lumbardy Database, to rule out inconsistencies and possible delays in transcriptions. This procedure has already been employed and validated in previous studies in Lumbardy, Italy [11, 30]. The limit date of June 30, 2018 was established for all patients for deaths, admissions, and exemptions. Causes of death, as well as exemptions and hospital admissions were coded according to ICD-10 codes. Full details of the procedures are reported elsewhere [11, 24, 30].

### Outcomes

Death rate and cause of death among patients with diabetes (surgical vs nonsurgical) and among patients without diabetes (surgical vs nonsurgical); exemptions and hospital admissions among patients with and without diabetes (surgical vs nonsurgical). Analysis of survival and of other outcomes was carried out on an intention-to-treat basis, with no consideration for LAGB removal.

### Statistical analysis

Data are shown as average values (± SD) for continuous variables or absolute numbers and frequencies for discrete variables. Continuous variables were compared with the Student’s t-test. Frequencies were compared with the Fisher exact test. Surgical and nonsurgical patients were matched (with and without diabetes separately) with no attempt to match patients of the whole cohort. Group matching was made for sex, BMI (± 5 kg/m^2^), age (± 10 years), for systolic (± 5 mmHg), and diastolic (± 5 mmHg) blood pressure. The median age of matched patients was 42 years, and the mean ages were 31.8 ± 6.43 and 51.8 ± 5.89, respectively. The proportion of dead patients was plotted through Kaplan–Meier curves, and differences in survival among subgroups were tested by the log-rank test. A multivariable analysis of risk factors for mortality was performed (Cox proportional hazards model), and used to plot Kaplan–Meier curves for surgery versus nonsurgical patients; age, median age, presence of diabetes, sex, systolic blood pressure, eGFR, and presence of CHD were entered a priori. Proportionality among the survival rates and attributable factors in the Cox model was assessed by plotting the log [− log (survival function)] versus time. Statistical analyses were performed with STATA 12.0 for MacIntosh.

### Power calculation and sample size

Being a retrospective study, power calculation and sample size were only calculated to understand if the study was meaningful. Due to previous papers dealing with long-term prevention of mortality, showing effectiveness of about 50% in comparison with non-surgery subjects [9, 10], given a power = 80% and an alfa error 0.05, it was calculated that 500 surgery subjects with 30 fatal events and 1000 nonsurgical subjects with 90 fatal events were required to detect significant differences in the outcomes [31, 32]. Similarly, given the high efficacy of bariatric surgeries in the long-term prevention of diabetes and of cancer, [33–35], we estimated that the occurrence of 100 exemptions in 500 bariatric surgery subjects and 300 exemptions in 1500 subjects undergoing dietary and medical treatment would be required to detect significant differences in the outcomes between the two groups [31, 32]. This manuscript was prepared following the guidelines of the STROBE statement [36] (Additional file 1).

## Results

The details of patients in the study were already published in a previous publication [11], and now appear in Additional file 2: Table S1. Observation period was 19.5 ± 1.87 years (13.34–23.5). Mortality rate was 2.6, 6.6, 10.1, and 13.4% in controls at 5, 10, 15, and 20 years, respectively; mortality rate was 0.8, 2.5, and 3.1, and 7.4% in LAGB patients at 5, 10, 15, and 20 years, respectively.

**Fig. 1:**
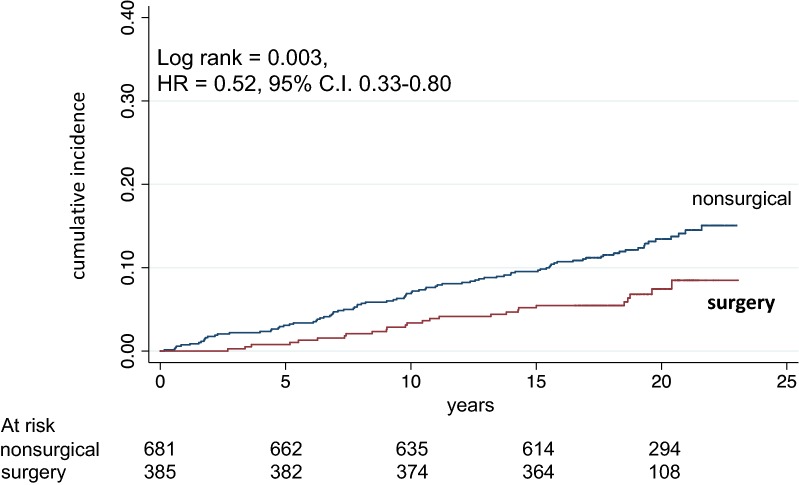
Mortality in surgical and in nonsurgical control patients, matched for age, sex, body mass index and blood pressure. Number of patients at risk is indicated. Years = since visit 1

**Fig. 2:**
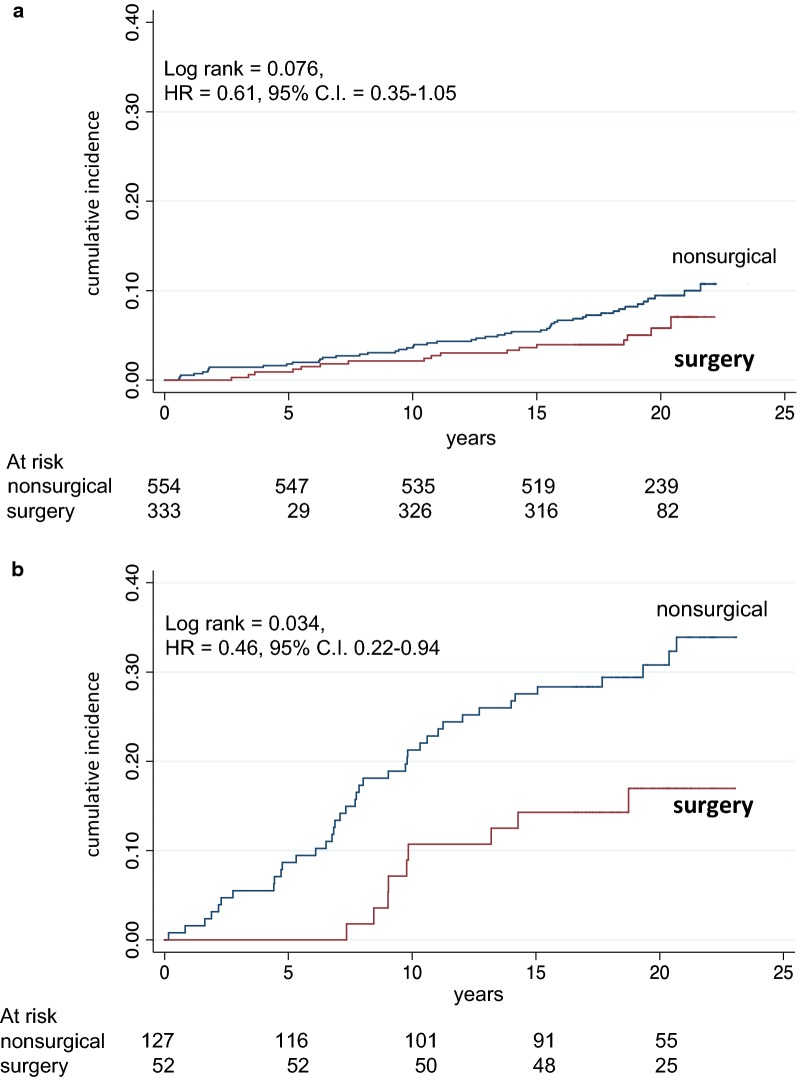
Mortality in surgical and in matched nonsurgical control patients divided into patients without (**a**) and with (**b**) diabetes. Number of patients at risk is indicated. Years = since visit 1

Figure 1 shows crude mortality curves in patients receiving LAGB as compared to controls receiving medical weight loss therapy, and Fig. 2a and b show crude mortality curves in patients without and with diabetes, respectively. The reduced mortality in surgical vs nonsurgical patients was significant in the whole cohort and in patients with diabetes, of borderline significance in patients without diabetes. During the first 5 years there were 4 deaths (1 above median age) in the surgery group and 18 deaths (17 above median age) in the nonsurgical group (NS). After exclusion of these patients, the HR was 0.32 (95% CI 0.15–0.69), (Log rank = 0.003).

**Fig. 3:**
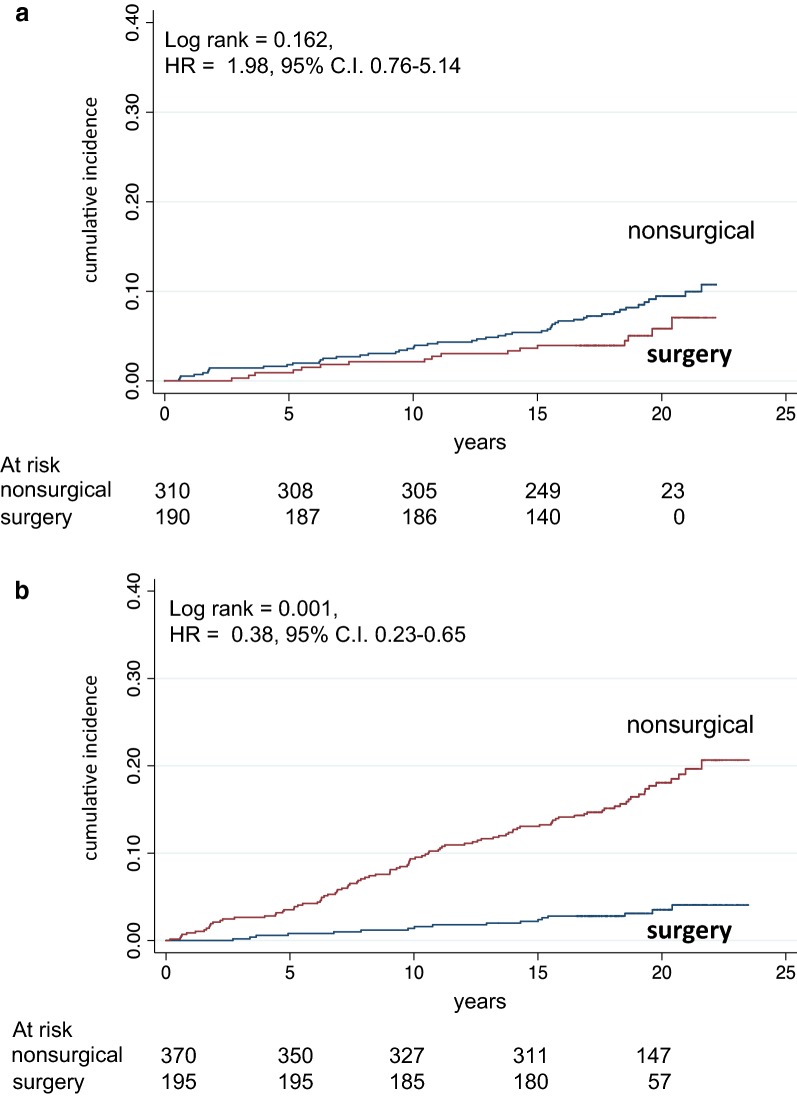
Mortality in surgical and in matched nonsurgical control patients divided according to median age (42 years): **a** below median age; **b** above median age. Number of patients at risk is indicated. Years = since visit 1

Figure 3a, b shows crude mortality curves in patients receiving LAGB as compared to controls receiving medical weight loss therapy, subdivided into aged < 42 years and aged > 42 years, respectively. The reduced mortality in surgical vs nonsurgical patients was significant in patients aged > 42 years, not significant in patients aged < 42 years. Table 1 shows causes of death in the whole cohort in the original study and in the follow-up study; causes of death were similar in the two observation periods, and the comparison between surgical vs nonsurgical patients had a reduced level of significance in the follow-up period, in agreement with the reduced overall effect on prevention of mortality.

**Table 1:** Causes of death in surgery and nonsurgical patients during the original study (observation period 13.9 ± 1.87 years, mean ± SD, 10) and in the follow-up study (observation period 19.5 ± 1.88 years)

Group	Original study		*p*	Follow-up study		*p*
	Surgery	Nonsurgical		Surgery	Nonsurgical	
CVD	5	22	0.001	8	32	0.029
Total non-CVD	7	43	0.001	18	58	0.019
Cancer	7	33	0.016	13	44	0.033
Liver	0	4	NS	2	4	NS
Lung	0	3	NS	1	4	NS
Infection	0	3	NS	1	4	NS
Endocrine	0	0	NS	0	1	NS
External	0	0	NS	1	1	NS
Total	12	65	0.001	26	90	0.001

Table 2 compares the 17 year and the 23 year effects of LAGB as opposed to medical weight loss therapy; the effect on reduced mortality decreases with time, while the effect on prevention of co-morbidities and the effect on prevention of hospital admissions increases with time.

**Table 2:** Comparison of mortality (HR with 95% CI), incident diseases, and hospital admissions in surgery and nonsurgical patients during the original study (observation period 13.9 ± 1.87 years, mean ± SD, 10) and in the follow-up study (observation period 19.5 ± 1.88 years)

	Original study	*p*	Follow-up study	*p*
Mortality	HR = 0.35, 95% CI 0.19–0.65	0.001	HR = 0.52, 95% CI 0.33–0.80	0.003
In non-DM	HR = 0.42, 95% CI 0.19–0.97	0.041	HR = 0.61, 95% CI = 0.35–1.05	0.076
In DM	HR = 0.34, 95% CI 0.13–0.87	0.025	HR = 0.46, 95% CI 0.22–0.94	0.034
Below median age	HR = 0.69, 95% CI 0.18–2.68	0.586	HR = 1.98, 95% CI 0.76–5.14	0.162
Above median age	HR = 0.29, 95% CI 0.14–0.58	0.001	HR = 0.38, 95% CI 0.23–0.65	0.001

	Surgery	Nonsurgical	*p*	Surgery	Nonsurgical	*p*
Incident diseases						
Diabetes	15	48	0.018	15	75	0.001
Arterial hypertension	42	107	0.028	47	174	0.001
CVD	10	17	NS	14	52	0.009
Renal diseases	0	3	NS	0	35	0.001
Liver diseases	8	18	NS	8	25	NS
Cancer	4	17	NS	10	35	0.01
Lung diseases	4	9	NS	4^a^	9^a^	NS
Metabolic diseases	4	13	NS	4^a^	13	NS
Total	87	232	0.001	102	418	0.001
Hospital admissions						
Diabetes	14	33	NS	14	120	0.001
CVD	34	69	NS	35	119	0.001
Liver disease	4	11	NS	6	28	0.028
Renal diseases	0	4	NS	0	8	NS
Cancer	9	22	NS	10	44	0.005
Lung diseases	5	18	NS	5^a^	18^a^	NS
Metabolic diseases	5	12	NS	5^a^	12^a^	NS
Muscular and bone diseases	17	28	NS	17^a^	28^a^	NS
Total	88	197	0.04	92	377	0.001

^a^When no new incident diseases of hospital admissions were recorded, data from the original study are indicated

Table 3 shows the clinical and metabolic effects of LAGB and medical weight loss therapy. The interval between baseline and follow-up data was 4.9 ± 3.63 years (mean ± SD), with no differences between surgery and nonsurgical patients. The effects were clearly different, with the noticeable exceptions of cholesterol (total, LDL-, and HDL-cholesterol).

**Table 3:** Variables evaluated at baseline and follow-up (4.9 ± 3.63 years)

	Surgery (n = 154)		*p*	Nonsurgical (n = 360)		*p*
	Baseline	Follow-up		Baseline	Follow-up	
Age (years)	41.0 ± 10.13	–	–	42.2 ± 12.94	–	–
BMI (kg/m^2^)	42.7 ± 4.62	36.7 ± 5.24	0.0001	39.1 ± 5.27	39.1 ± 6.16	0.5047
Blood glucose (mg/dL)	108.6 ± 39.36	97.6 ± 32.09	0.0001	103.1 ± 27.40	103.6 ± 30.47	0.4452
Hba1c (%)	6.0 ± 1.35	5.5 ± 1.01	0.0001	7.6 ± 2.51	6.7 ± 1.64	0.1055
Systolic BP (mmHg)	133.8 ± 14.59	127.2 ± 12.38	0.0001	132.4 ± 13.36	132.8 ± 8.76	0.9090
Diastolic BP (mmHg)	83.9 ± 9.55	77.8 ± 8.68	0.0001	78.65 ± 9.48	78.8 ± 10.43	0.5203
Heart rate (bpm)	78.2 ± 5.95	55.7 ± 28.89	0.0304	71.2 ± 6.46	75.8 ± 10.56	0.1280
EGFR (mL/min/1.73 m^2^)	107.3 ± 29.07	99.3 ± 23.34	0.0010	85.4 ± 21.54	90.2 ± 18.83	0.0811
Cholesterol (mg/dL)	207.7 ± 43.77	203.2 ± 36.77	0.0685	210.5 ± 32.68	198.8 ± 37.43	0.0272
LDL-cholesterol (mg/dL)	130.6 ± 40.50	124.44 ± 32.60	0.0250	135.4 ± 35.07	119.3 ± 34.81	0.0214
HDL-cholesterol (mg/dL)	50.1 ± 13.52	54.6 ± 13.87	0.0001	48.4 ± 11.82	51.29 ± 12.29	0.0298
Triglycerides (mg/dL)	140.4 ± 76.49	122.2 ± 66.93	0.0001	139.2 ± 68.34	130.8 ± 52.72	0.1735
AST (U/L)	23.7 ± 11.86	21.5 ± 8.55	0.0146	23.6 ± 10.41	24.5 ± 7.26	0.2830
ALT (U/L)	31.3 ± 21.14	24.2 ± 15.39	0.0001	32.9 ± 27.64	30.4 ± 15.19	0.2568

Mean ± SD

*BMI* body mass index, *HbA1c* glycated hemoglobin, *EGFR* estimated glomerular filtration rate (mL/min/1.73 m^2^), *AST* aspartate transaminase, *ALT* alanine transaminase

Table 4 shows univariate and multivariate analysis of risk factors for mortality in the current study as compared with the original study, and indicates that risk factors considered in the original study maintained their value in the follow-up study.

**Table 4:** Univariate and multivariable analysis of risk factors for mortality (Cox proportional hazards model) in the whole sample Hazard ratios (HR, with 95% CI) and standard errors are indicated, together with effect (z) and significance level

	HR	S.E.	Z	*p*	95% CI
Univariate analysis					
Surgery	0.52 (0.35)	0.12 (0.11)	− 2.94 (− 3.33)	0.003 (0.001)	0.33–0.81 (0.19–0.65)
Age > 42 years	5.53 (7.15)	1.45 (2.43)	6.52 (5.81)	0.001 (0.001)	3.31–9.26 (3.68–13.91)
Female sex	0.53 (0.39)	0.10 (0.09)	− 3.27 (− 4.02)	0.001 (0.001)	0.36–0.78 (0.25–0.62)
Coronary heart disease	4.98 (4.67)	1.35 (1.52)	5.94 (4.73)	0.001 (0.001)	2.93–8.47 (2.47–8.86)
Diabetes	3.94 (5.71)	0.74 (1.31)	7.29 (7.61)	0.001 (0.001)	2.73–5.70 (3.54–8.94)
Multivariable analysis					
Surgery	0.51 (0.41)	0.12 (0.13)	− 2.95 (− 2.82)	0.003 (0.005)	0.33–0.80 (0.22–0.76)
Age > 42 years	4.31 (4.35)	1.21 (1.57)	5.21 (4.08)	0.001 (0.001)	2.49–7.45 (2.15–8.82)
Female sex	0.53 (0.39)	0.10 (0.09)	− 3.25 (− 4.10)	0.001 (0.001)	0.36–0.78 (0.25–0.61)
Coronary heart disease	2.83 (2.51)	0.78 (0.83)	3.77 (2.75)	0.001 (0.006)	1.65–4.87 (1.31–4.81)
Diabetes	2.65 (3.11)	0.53 (0.75)	4.86 (4.69)	0.001 (0.001)	1.79–3.93 (1.93–4.99)

In brackets values observed in the original study [10]

## Discussion

To our knowledge, this study represents the longest follow-up evaluation of patients undergoing LAGB, a bariatric surgery, in comparison with patients receiving weight loss medical treatment. With its up to 23 years duration of observation, this study adds about 6 years to our previous study, in the same cohort, studied in the same way. The main finding, in comparison with our previous study [11], is the somehow reduced effect on prevention of long-term mortality in comparison with our previous study; in contrast, the preventive effect of surgery on incident diseases increases, and the preventive effect of surgery on hospital admissions increases. Therefore, it appears that the beneficial effect of LAGB continues up to 23 years, even with some differences; the effect on mortality decreases, even it is still significant, while the effect on general health status continues, and increases. Overall, as recently confirmed by recent 4–5 year studies performed through various surgical techniques (LGB, RYGB, LSG) [23], our data confirm that bariatric surgery is associated with lower mortality compared to medical weight loss treatment [9, 10]; also prevention of co-morbidities, especially diabetes mellitus, is possible for prolonged periods [27, 33, 37, 38].

A greater effect on mortality in patients with diabetes than in patients without diabetes has already been reported [12], leading to the interpretation that the benefit is greater in more compromised patients. There are no explanations for these differences, though it seems reasonable to assume that the aging process dilutes the preventive effect of LAGB on mortality. In the swedish obesity study (SOS study) [37] it was observed that the preventive effect of surgery on incident co-morbidities increases with duration of follow-up (from 2 to 10 years); our data support these findings, even though the observation periods of the two studies are quite different. However, we observed that the effect of surgery depends on age, i.e. it is significant for patients above median age (42 years in this cohort), not in younger patients. This confirms what was already observed by us and by others, using different bariatric techniques [5, 8, 11, 39]; in the SOS Study, patients aged < 37 years were intentionally excluded because of the low mortality of patients with obesity in young age [4].

This study has strengths and limitations; the main strength lies in the prolonged observation period of the same cohort, evaluated with the same approach; also, due to the methods employed, no patient was lost to follow-up. In addition, we had detailed description of causes of death of all patients, of incident diseases, of hospital admissions. More, we had the possibility to observe clinical and metabolic variables in a fair proportion of patients after a mean period of 5 years, and we could observe a significant different effect of surgery vs medical weight loss treatment. Obesity, and especially visceral obesity, favor development of cardiovascular disease in type 2 diabetes [40], and both type 2 diabetes and obesity predict all-cause mortality [41, 42]; the present results indicate that LAGB, able to induce weight loss and to prevent diabetes, prevents mortality through improvement of the general health status [43]. Finally, as reported above, we confirmed a significant age-related effect on prevention of mortality, in agreement with previous studies [5, 8, 11, 39].

The main limitation lies in the retrospective nature of the study; the second limitation is that the study was not randomized, but at the time this study was conceived, randomization was deemed unethical, so that prospective studies could not be performed. The fact that several patients refused surgery for multiple reasons might represent a selection bias; however, it should be emphasized that in the years 1995–2001 evidence of benefits of bariatric surgery were still limited. Also, during the first 5 years there were 4 deaths (1 above median age) in the surgery group and 18 deaths (17 above median age) in the nonsurgical group (NS); we have no explanation for a higher number of early deaths in both groups is higher than in previous papers [10], but differences in different cohorts can occur. The fourth limitation is in the sample size. The fifth limitation is represented by the fact that the use of of LAGB is declining, so that some people argue LAGB should be abandoned; actually, LAGB is still performed in a significant proportion of patients with obesity. The last limitation is that our results can not be generalized to all bariatric procedures, also because there are no studies of similar duration performed with other bariatric techniques.

## Conclusion

The preventive effect of LAGB on mortality is maintained up to 23 years, even with a decreased efficacy, while the preventive effect of LAGB on incident diseases and on hospital admissions increases with time. These data indicate that the beneficial effects of LAGB is long lasting.

## Additional files

**Additional file 1.** Strobe statement.

**Additional file 2: Table S1.** Subjects in the study.

## Abbreviations

ALT: alanine transferase
AST: aspartate transferase
BIBP: biliointestinal bypass
BPD: biliopancreatic diversion
BMI: body mass index
CV: cardiovascular
CI: confidence intervals
CHD: coronary heart disease
RYGB: roux-en-y gastric bypass
eGFR: estimated glomerular filtration rate
LAGB: gastric band
HbA1c (%): glycated hemoglobin
HR: hazard ratio
ASL: health districts
NHS: National Health System
LSG: laparoscopic sleeve gastrectomy
SOS study: Swedish obesity study
VGB: vertical banded gastroplasty
