# Supplementary material for: A 23-year study of mortality and development of co-morbidities in patients with obesity undergoing bariatric surgery (laparoscopic gastric banding) in comparison with medical treatment of obesity
Source: Cardiovasc Diabetol. 2018 Dec 29;17:161. doi: 10.1186/s12933-018-0801-1 (PMC6311074; doi:10.1186/s12933-018-0801-1)
Supplement: Supplementary file 1 — Additional file 1. Strobe statement. [file 12933_2018_801_MOESM1_ESM.doc]

A 23-year study of mortality and development of co-morbidities in patients with obesity undergoing bariatric surgery (laparoscopic gastric banding) in comparison with medical treatment of obesity.

STROBE Statement—checklist of items that should be included in reports of observational studies

|  | Item No | Recommendation |
| --- | --- | --- |
| **Title and abstract** | 1 | **See Page 1** Indicate the study’s design with a commonly used term in the title or the abstract |
|  |  | **See Page 1** Provide in the abstract an informative and balanced summary of what was done and what was found |
| Introduction | | |
| Background/rationale | 2 | **See Page 2** Explain the scientific background and rationale for the investigation being reported |
| Objectives | 3 | **See Page 2** State specific objectives, including any prespecified hypotheses |
| Methods | | |
| Study design | 4 | **See Page 2 / 3** Present key elements of study design early in the paper |
| Setting | 5 | **See Pages 2 / 3** Describe the setting, locations, and relevant dates, including periods of recruitment, exposure, follow-up, and data collection |
| Participants | 6 | (*a*) *Cohort study*—Give the eligibility criteria, and the sources and methods of selection of participants. Describe methods of follow-up  **See Pages 2 / 3** |
|  |  | (*b*) *Cohort study*—For matched studies, give matching criteria and number of exposed and unexposed  **See Pages 3 / 4** |
| Variables | 7 | Clearly define all outcomes, exposures, predictors, potential confounders, and effect modifiers. Give diagnostic criteria, if applicable **See Page 4** |
| Data sources/ measurement | 8* | **See Pages 3/4** For each variable of interest, give sources of data and details of methods of assessment (measurement). Describe comparability of assessment methods if there is more than one group |
| Bias | 9 | **See Pages 3 / 4** Describe any efforts to address potential sources of bias |
| Study size | 10 | **See Pages 4 / 5** Explain how the study size was arrived at |
| Quantitative variables | 11 | **See Page 4** Explain how quantitative variables were handled in the analyses. If applicable, describe which groupings were chosen and why |
| Statistical methods | 12 | **See Pages 4** (*a*) Describe all statistical methods, including those used to control for confounding |
|  |  | **See Pages 4**  (*b*) Describe any methods used to examine subgroups and interactions |
|  |  | **No Applicable** (*c*) Explain how missing data were addressed |
|  |  | **No Applicable** (*d*) *Cohort study*—If applicable, explain how loss to follow-up was addressed |
|  |  |  |

Continued on next page

| Results | | |
| --- | --- | --- |
| Participants | 13* | **See Page 4/5** (a) Report numbers of individuals at each stage of study—eg numbers potentially eligible, examined for eligibility, confirmed eligible, included in the study, completing follow-up, and analysed |
|  |  | **No Applicable** (b) Give reasons for non-participation at each stage |
|  |  |  |
| Descriptive data | 14* | **See Pages 5 / 6** (a) Give characteristics of study participants (eg demographic, clinical, social) and information on exposures and potential confounders |
|  |  | **No Applicable** (b) Indicate number of participants with missing data for each variable of interest |
|  |  | (c) *Cohort study*—Summarise follow-up time (eg, average and total amount) **No Applicable** |
| Outcome data | 15* | *Cohort study*—Report numbers of outcome events or summary measures over time **See Page 4 / 5** |
| Main results | 16 | **See Pages 4 / 5 and figures 1** (*a*) Give unadjusted estimates and, if applicable, confounder-adjusted estimates and their precision (eg, 95% confidence interval). Make clear which confounders were adjusted for and why they were included |
|  |  | **See Pages 4 / 5** (*b*) Report category boundaries when continuous variables were categorized |
|  |  | **See Pages 4 / 5** (*c*) If relevant, consider translating estimates of relative risk into absolute risk for a meaningful time period |
| Other analyses | 17 | **See Pages 4 / 5 and Table 3** Report other analyses done—eg analyses of subgroups and interactions, and sensitivity analyses |
| Discussion | | |
| Key results | 18 | **See Page 5 / 6** Summarise key results with reference to study objectives |
| Limitations | 19 | **See Page 5 / 6** Discuss limitations of the study, taking into account sources of potential bias or imprecision. Discuss both direction and magnitude of any potential bias |
| Interpretation | 20 | **See Page 5 / 6** Give a cautious overall interpretation of results considering objectives, limitations, multiplicity of analyses, results from similar studies, and other relevant evidence |
| Generalisability | 21 | **See Page 5 / 6** Discuss the generalisability (external validity) of the study results |
| Other information | | |
| Funding | 22 | **See Page 6 / 7** Give the source of funding and the role of the funders for the present study and, if applicable, for the original study on which the present article is based |

*Give information separately for cases and controls in case-control studies and, if applicable, for exposed and unexposed groups in cohort and cross-sectional studies.

**Note:** An Explanation and Elaboration article discusses each checklist item and gives methodological background and published examples of transparent reporting. The STROBE checklist is best used in conjunction with this article (freely available on the Web sites of PLoS Medicine at http://www.plosmedicine.org/, Annals of Internal Medicine at http://www.annals.org/, and Epidemiology at http://www.epidem.com/). Information on the STROBE Initiative is available at www.strobe-statement.org.
