# Supplementary material for: A 23-year study of mortality and development of co-morbidities in patients with obesity undergoing bariatric surgery (laparoscopic gastric banding) in comparison with medical treatment of obesity
Source: Cardiovasc Diabetol. 2018 Dec 29;17:161. doi: 10.1186/s12933-018-0801-1 (PMC6311074; doi:10.1186/s12933-018-0801-1)
Supplement: Supplementary file 2 — Additional file 2: Table S1. Subjects in the study. [file 12933_2018_801_MOESM2_ESM.doc]

**Table S1**

Subjects in the study in patients matched (with and without diabetes, separately) for sex, age, BMI, systolic and diastolic

blood pressure (surgery vs nonsurgical)

| **Groups** | **1** | **3** | **2** | **4** |
| --- | --- | --- | --- | --- |
|  | **Diabetes, surgery** | **Diabetes, no-surgery** | **No-diabetes, surgery** | **No-diabetes, nonsurgical** |
| **Patients (M/W)** | 52 (15/37) | 127 (36/91) | 333 (78/255) | 554 (136/418) |
| **Age (y)** | 49.9 ± 5.25 | 51.9 ± 8.61 | 39.2 ± 10.37 | 40.2 ± 12.03 |
| **BMI (kg/m^2^)** | 43.0 ± 3.98 | 41.9 ± 6.31 | 41.1 ± 5.36 | 40.9 ± 7.31 |
| **Systolic BP (mmHg)** | 142.0 ± 12.29 | 148.0 ± 22.46 | 134.9 ± 16.48 | 134.4 ± 16.74 |
| **Diastolic BP (mmHg)** | 85.3 ± 5.95 | 85.9 ± 11.28 | 82.3 ± 10.41 | 82.8 ± 10.42 |
| **Heart rate (bpm)** | 80.9 ± 15.30 | 77.4 ± 13.64 | 75.6 ± 4.46 | 70.4 ± 4.72 |
| **Hypertension** | 15 | 53 | 77 | 132 |
| **Creatinine (umol/L)** | 74.4 ± 21.95 | 77.3 ± 25.26 | 74.6 ± 16.14 | 76.7 ± 19.84 |
| **EGFR (ml/min/1.73m^2^)** | 88.8 ± 20.19 | 88.0 ± 24.87 | 92.1 ± 26.13 | 87.8 ± 18.67 |
| **FBG (mg/dl)** | 169.7 ± 60.56 | 185.3 ± 62.55 | 92.4 ± 12.69 | 94.7 ± 12.19 |
| **DM drug treatment** | 6 (1) | 27 (2) |  |  |
| **HbA1c (%)** | 7.2 ± 2.19 | 8.1 ± 1.88 | 5.7 ± 1.17 | 5.6 ± 1.25 |
| **Total Cholesterol (mg/dl)** | 218.7 ± 43.74 | 219.4 ± 55.33 | 210.4 ± 43.26 | 212.0 ± 98.3 |
| **HDL-cholesterol (mg/dl)** | 50.3 ± 14.71 | 46.8 ± 13.46 | 51.1 ± 12.94 | 50.0 ± 15.10 |
| **LDL-cholesterol (mg/dl)** | 147.6 ± 38.43 | 144.4 ± 45.23 | 136.0 ± 37.24 | 140.2 ± 99.26 |
| **Triglycerides (mg/dl)** | 159.3 ± 80.06 | 208.8 ± 198.23 | 127.5 ± 70.62 | 135.3 ± 68.88 |
| **AST (U/L)** | 30.9 ± 22.28 | 30.6 ± 22.30 | 22.8 ± 10.20 | 24.6 ± 12.44 |
| **ALT (U/L)** | 42.3 ± 31.1 | 44.6 ± 44.93 | 31.2 ± 22.70 | 33.9 ± 22.67 |
| **Retinopathy** | 1 | 7 |  |  |
| **CHD** | 0 | 13 * | 4 | 22 * |

Mean ± SD or absolute frequencies

BP blood pressure, eGFR estimated glomerular filtration rate, FBG fasting blood glucose, CHD coronary heart disease

* p < 0.05 surgery vs nonsurgical

(1) 5 patients on metformin, 1 on insulin

(2) 3 patients on sulphanylureas, 13 patients on, 11 on sulphanylureas + metformin
